# Increased Awakenings From Non-rapid Eye Movement Sleep Explain Differences in Dream Recall Frequency in Healthy Individuals

**DOI:** 10.3389/fnhum.2019.00370

**Published:** 2019-10-15

**Authors:** Mariza van Wyk, Mark Solms, Gosia Lipinska

**Affiliations:** UCT Sleep Sciences, Department of Psychology, University of Cape Town, Cape Town, South Africa

**Keywords:** dream recall frequency, non-rapid eye movement sleep, rapid eye movement sleep, dreaming, sleep architecture, REM density

## Abstract

**Background:**

Dreaming is a universal experience, yet there is considerable inter-individual variability in dream recall frequency (DRF). One dominant model, the “arousal-retrieval” model, posits that intra-sleep wakefulness is required for dream traces to be encoded into long-term storage, essentially proposing that a better *memory* for dreams underlie increased DRF. A recent study utilizing polysomnography combined with an event-related potentials paradigm, provides direct support for this model by demonstrating increased intra-sleep wakefulness in a healthy population by comparing high frequency recallers (HFRs) and low frequency recallers (LFRs). Another study by the same group demonstrated increased regional cerebral blood flow in regions associated with dream production, supporting the premise that HFRs also may *produce* more dreams.

**Hypotheses:**

This study investigated the profile of nocturnal awakenings and dream production in healthy HFRs and LFRs. Hypothesis (1a): HFRs will spend significantly more time awake after sleep onset; (1b): HFRs will experience significantly more awakenings across the night, and from rapid eye movement (REM) sleep in particular; (2) HFRs will have significantly higher rates of dream production across the night as measured by REM density.

**Methods:**

We studied two groups of healthy adults: HFRs (*n* = 19) and LFRs (*n* = 17) who underwent polysomnographic recordings on two non-consecutive nights.

**Results:**

Hypothesis (1a) was confirmed: HFRs spent significantly more time awake after sleep onset. Hypothesis (1b) was partially confirmed: HFRs experienced significantly more awakenings across the night; however, awakenings from REM sleep were comparable. Interestingly, HFRs had significantly more awakenings, as well as a higher number of longer awakenings, from non-rapid eye movement (NREM) stage 2 sleep. Hypothesis (2) was not confirmed: There was no significant difference in rates of REM density between groups.

**Conclusion:**

This is the first study to provide evidence that awakenings from NREM 2 sleep might underlie increased DRF in HFRs. This finding coupled with null findings in relation to REM sleep variables, support the premise that inter-individual variability in DRF cannot be ascribed to differences in REM sleep parameters in healthy individuals. Instead, the data indicates that awakenings from NREM sleep is of particular importance in relation to DRF in a healthy population.

## Introduction

Dream recall rates vary considerably between individuals (Schredl et al., 2007). Multiple models have been developed in an attempt to explain this variability (Freud, 1958; Schonbar, 1965; Cohen and Wolfe, 1973; Cohen and MacNeilage, 1974). Among these, the arousal-retrieval model (Koulack and Goodenough, 1976) is supported by reliable empirical evidence (For a review, see Schredl, 1999; Schredl et al., 2003a,b).

This model proposes a mechanism for how dream content is transferred from short-term consciousness to long-term memory storage. The model assumes that traces are not encoded during the dreaming process itself. One possible explanation for the lack of encoding could be related to the substantial deactivation of the prefrontal cortex during both non-rapid eye movement (NREM) and rapid eye movement (REM) sleep (Muzur et al., 2002; Nir and Tononi, 2010; Mutz and Javadi, 2017). The prefrontal cortex is essential for executive functions involved in the encoding of complex content. Therefore, according to the model, a period of wakefulness is necessary to enable long-term storage of short-term dream content. If this occurs, subsequent retrieval from long-term storage is enabled.

Several lines of research provide some support for the arousal-retrieval model of dream recall. Studies of individuals who experience frequent arousals during sleep, due for example to insomnia or sleep apnea, show increased dream recall frequency (DRF; Schredl, 1999, 2009, 2010; Schredl et al., 1999). However, DRF in individuals with abnormal sleep may be confounded by their sleep pathologies, while there might be factors other than arousals contributing to increased DRF in this population group.

Two recent studies managed to circumvent at least one major confound evident in earlier research by recruiting healthy participants. De Gennaro et al. (2010), utilizing polysomnography, recruited 40 individuals to investigate the effect of a single night of total sleep deprivation on DRF the morning following recovery sleep. The authors found a near-complete abolition of morning dream recall. They propose that one explanation relates to the significant decrease in the number of awakenings on the recovery night, a finding they propose to be consistent with the arousal-retrieval model. Another study utilized a design where high frequency recallers (HFRs; *n* = 18) are compared directly to low frequency recallers (LFRs; *n* = 18; Eichenlaub et al., 2014a). They investigated various sleep parameters, including arousals and awakenings. A significant difference with regard to “intra-sleep wakefulness” was found, i.e., individuals with high rates of dream recall spent significantly more time awake following sleep onset.

This study provided critical evidence for the arousal-retrieval model; however, it should be interpreted with caution as questions remain as to whether mechanisms other than (or in addition to) arousal-retrieval cause higher DRF in HFRs. For example, it may be precisely because HFRs are alerted to their dreams via awakening that they report a higher frequency of dreams in the first place. Alternatively, HFRs may actually produce more dreams, which, in combination with increased wakefulness, results in higher dream recall.

To investigate the latter possibility, a study by the same group recruited healthy HFRs (*n* = 21) and LFRs (*n* = 20) and measured regional cerebral blood flow (rCBF) during both sleep and wakefulness (Eichenlaub et al., 2014b). The study found that (a) compared to LFRs, HFRs showed significantly increased rates of rCBF in the temporo-parietal junction (TPJ) during NREM stage 3 sleep (NREM3), REM sleep, and wakefulness, and (b) significantly increased rCBF in the medial prefrontal cortex (mPFC) during REM sleep and wakefulness. Based on this, the authors propose that the TPJ and mPFC play an important role not only in relation to dream recall during wakefulness, but also in the dreaming process itself. The study by Marzano et al. (2011) also gives credence to the importance of frontal and temporo-parietal areas in the dreaming process. They investigated possible neurophysiological correlates associated with successful recall upon awakening during REM and NREM 2 sleep. The authors found that an increase in frontal theta activity during REM sleep, and lower alpha activity in the right temporo-parietal areas during NREM2 sleep, were associated with subsequent successful dream recall. Authors from both studies note that lesion studies provide support for the important, yet not exclusive, role of the TPJ and mPFC in dream production. These studies demonstrate that complete or near-complete cessation of dreaming frequently occurs with damage to the mPFC and TPJ (Murri et al., 1985; Doricchi and Violani, 1992; Solms, 1997). Overall, these results suggest that HFRs not only have increased intra-sleep wakefulness, which promotes dream recall, but may also have increased dream production.

However, measuring dream production directly remains a methodological challenge. This is because subjective dream recall does not necessarily produce reliable estimates of actual dream frequency (Schredl et al., 2003b; Parke and Horton, 2009; Kahan and LaBerge, 2011). An alternative index of dream production is REM density (the frequency of rapid eye movements during REM sleep). Studying dream production via REM sleep parameters serves as a reasonable starting point as REM sleep typically yields the highest rates of dream recall (up to 90%) compared to NREM sleep (10–54%; for reviews, see Stickgold et al., 1994; Nielsen, 2000, 2004; Schredl et al., 2007). Importantly, there is empirical support for using REMs to index the occurrence of dreaming: REMs function as a physiological correlate of ponto-geniculo-occipital (PGO) wave activity, while PGO waves sub-serve the occurrence of dream imagery (Pace-Schott, 2005; Miyauchi et al., 2009; Leclair-Visonneau et al., 2010; Desseilles et al., 2011). Therefore, based on PGO activity serving as a common underlying mechanism, the incidence of REMs can be associated with the occurrence of dream imagery during REM sleep.

To our knowledge, there is only one study comparing REM density in healthy HFRs and LFRs (Vallat et al., 2017a). This study compared 18 HFRs with 18 LFRs and showed that there was no significant difference in REM density between the two groups. Therefore, the question as to whether HFRs not only *report* more dreams but also *produce* more dreams warrants additional consideration.

The current study has two aims. The first aim is to investigate whether HFRs and LFRs differ in their profile of nocturnal awakenings (including both time spent awake and the number of awakenings, based on measures from the whole night and from REM sleep in particular). The second aim is to investigate whether HFRs produce more dreams across the night when compared to LFRs.

The following hypotheses were tested:

Hypothesis 1:

(a) High frequency recallers will spend significantly more time awake after sleep onset compared to LFRs.
(b) High frequency recallers will experience a significantly increased number of awakenings across the night, and from REM sleep in particular, compared to LFRs.

Hypothesis 2:

High frequency recallers will exhibit significantly higher rates of REM density across the night compared to LFRs.

## Materials and Methods

### Participants

We recruited male and female HFRs and LFRs using a university population. To classify them, potential participants were asked – without being informed that DRF was the primary criterion of inclusion – about their DRF, using the definition provided by Eichenlaub et al. (2014a, b) in similar research: “If a dream is defined as a long and bizarre story, an image that vanishes rapidly, or a feeling of having dreamed, on average, how many mornings per week over the last couple of months did you wake up with a dream in mind?”

Based on their responses, participants recalling more than three dreams per week were classified as HFRs and those recalling less than two dreams per month were classified as LFRs. Only individuals falling in one of these two categories were considered for participation in the study (Eichenlaub et al., 2014a, b).

Screening occurred in two phases: an online survey phase and a face-to-face clinical interview. During the online screening phase, participants completed questions related to demographics, medical and psychiatric history, sleep quality, unusual sleep experiences (e.g., sleep paralysis), medication use, and DRF. Questions regarding DRF were embedded in the middle of the online screener. This was done in order to minimize reported DRF bias which can result from pre-existing attitudes toward dreams (see the meta-analysis by Beaulieu-Prévost and Zadra, 2007).

A total of 2041 individuals responded to the online survey, with 1591 successfully completing it. Of the 1591 individuals who completed the survey, 1051 (68%) were excluded due to not meeting the DRF criteria. Of the remaining individuals, 370 potential participants met the criteria for HFR, and 170 met the criteria for LFR. Based on results from the online survey pertaining to medical, psychiatric, and sleep quality data, 348 potential participants were excluded. Of the remaining 192 eligible potential participants, 138 declined an invitation to advance to the next screening phase. 56 potential participants agreed to the second screening phase which entailed a face-to-face clinical interview. The interview probed potential participants’ psychiatric and intellectual functioning in greater detail. Based on results from the clinical interview, 20 potential participants were excluded. The final sample (*N* = 36) consisted of 19 HFR individuals and 17 LFR individuals, a sample size consistent with other studies in this field (Eichenlaub et al., 2014a, b). The HFR group contained 11 females and 8 males, while the LFR group contained 9 males and 8 females.

Participants were excluded from participation if they: (a) were below the age of 20 or over the age of 40, (b) had any medical or neurological condition that could influence the outcomes of the study, (c) had a current and/or past history of a sleep disorder, (d) had a current and/or past history of a psychiatric disorder, (e) used sleeping pills, sedative medication, psychoactive medication or any other medication that might affect dreaming, (f) had a past and/or current history of alcohol or substance abuse/dependence, (g) were pregnant, or (h) had reduced cognitive ability. Literature shows that these factors have an independent relationship with sleep and/or dreaming (Lee, 1998; Blackman, 2000; Irwin et al., 2000; Nielsen and Stenstrom, 2005; Schredl, 2009; Pagel, 2010; Schredl et al., 2013; Skancke et al., 2014).

### Materials and Apparatus

#### Screening Measures

##### Online screening

The (a) *Michigan Alcoholism Screening Test* (MAST; Selzer, 1971) was used to exclude participants that were alcohol dependent (MAST > 4), (b) the *Pittsburgh Sleep Quality Index* (PSQI; Buysse et al., 1989) was used to exclude participants with poor sleep quality (PSQI > 5), and (c) the *Beck Depression Inventory*, 2nd Edition (BDI-II; Beck et al., 1996) was used to exclude participants with depressive symptomatology (BDI-II > 13).

##### Face-to-face screening

The *Mini International Neuropsychiatric Interview* (Version 5.0.0; MINI; Sheehan et al., 1998) is a structured diagnostic interview that was used to exclude participants with any major psychiatric disorders contained in the Diagnostic and statistical manual of mental disorders (DSM-V; American Psychiatric Association, 2013). These include depression, bipolar disorder, posttraumatic stress disorder, alcohol use/dependence, substance use/dependence, and obsessive-compulsive disorder. This measure was also used to cross-validate results obtained via the MAST and BDI-II. That is, participants who scored adequately on the online screening measures but screened positive for alcohol use/dependence and depression on the MINI were excluded from participation.

*The Shipley-2 IQ Test* (Kaya et al., 2012) is a revised and re-standardized test that provides a robust measure of both crystallized and fluid intelligence. It was used to exclude participants with an IQ < 80.

#### Experimental Measure

##### Polysomnography

Two 16-channel Nihon Kohden Neurofax EEG900 electroencephalographs that were adapted for research recorded objective measures of sleep. Polysomnography includes: electroencephalography (EEG) which measures brain activity, electrooculography (EOG) which monitors eye movements, chin electromyography (EMG) which monitors muscle tone, and electrocardiography (ECG) which measures heart rate.

A bipolar montage was used with the following bipolar derivations: F3-C3, C3-P3, P3-O1, and F4-C4, C4-P4, P4-O2. This was combined with a referential montage utilizing F3-A2, C3-A2, O1-A2, and F4-A1, C4-A1, O2-A1 derivations. A combination approach was chosen in order to ensure the integrity of the records. Standard filters for sleep recordings were used for the EEG and EOG (0.5–35 Hz), EMG (10–70 Hz), and ECG (1–70 Hz) as recommended by AASM guidelines (American Academy of Sleep Medicine, 2015).

### Study Procedure

All study procedures complied with the Declaration of Helsinki and we obtained ethical clearance from the ethical review boards of both the Psychology Department and the Faculty of Humanities. Before commencing, all participants completed an informed consent form.

Following screening, eligible participants who provided consent entered the first phase of the study, the adaptation night. During this phase, participants spent a night in the sleep laboratory in order to familiarize themselves with the equipment and the sleeping environment. Recordings started at 23:00 h for a duration of 7.5 h. Each participant completed a Most Recent Dream Form (Domhoff and Schneider, 1998) upon awakening.

During the second phase, participants spent a non-consecutive night in the sleep laboratory where they were connected to the polysomnography and allowed to sleep for 7.5 h. Upon awakening participants again completed the Most Recent Dream Form and were remunerated for their time.

### Statistical Analysis

Sleep data were scored according to the latest specification provided by the American Academy of Sleep Medicine (2015) to derive the following variables of interest: sleep onset latency (SOL; time spent falling asleep); total awakenings from sleep, as well as awakenings from (a) NREM 2 sleep, (b) NREM 3 sleep, and (c) REM sleep; percentage of time spent awake after sleep onset (WASO%); sleep efficiency (SE); percentage of sleep spent in REM sleep (REM%); REM sleep latency; and percentage of sleep spent in each of NREM 1–3 (NREM 1%; NREM 2%; and NREM 3%). All inferential statistics were conducted using SPSS software, version 22, with alpha set at 0.05 for decisions regarding statistical significance. Where the data violated parametric assumptions, we used non-parametric statistics instead.

### REM Density Analysis

Within each REM sleep stage, REM density was calculated through a process, which was based on a method employed by Stanford’s Centre for Sleep Sciences (Moore and Mignot, 2015). Each electrooculograph (EOG) channel was 50 Hz notch filtered; bandpass filtered from 0.3 to 30 Hz and then down-sampled to 100 Hz. A double-threshold eye movement detection method was employed to mark REM events: the high threshold was set at 30 uV and the low threshold at 10 uV; multiple eye movements within 0.05 s were merged into one movement and the minimum duration of an eye movement was set at 0.1 s.

Each REM stage was then divided into 30 s epochs, and each of those epochs was then further sub-divided into 2 s mini-epochs. Each of these 2 s mini-epochs was inspected for the presence of eye movements. There needed to be at least one eye movement in each 2 s mini-epoch to conclude that an eye movement had taken place within that mini-epoch; any more than one eye movement was still recorded as a single movement within a mini-epoch.

A REM density value was then calculated for each 30 s epoch as the percentage of 2 s mini-epochs that contained at least one REM in either EOG channel. Following this, each EOG epoch was further also qualitatively inspected for artifact, and where artifact was identified, the corresponding REM density values were disregarded. REM density was thus calculated for every 30 s epoch of each REM stage, for each of the two EOG channels.

The total REM density value for each participant was calculated as part of a two-step process. Firstly, the mean REM density percentage was calculated for each REM cycle. This was achieved by considering each REM cycle separately and averaging all the values within that specific REM sleep cycle in order to obtain a single value for each REM cycle. Secondly, the total REM density value per participant was derived from averaging the final values of all the REM sleep cycles.

## Results

Table 1 shows the sociodemographic and screening outcomes of the 36 individuals who participated in the study. The two groups were matched in terms of age, sex, highest level of education (HLOE), Shipley 2 IQ score, depressive symptomatology scores, subjective sleep quality, and alcohol abuse and dependence.

**TABLE 1:** Sociodemographic and screening outcomes of the current sample (*N* = 36).

	**Group**				
	**HFR**	**LFR**			
**Variable**	**(*n* = 19)**	**(*n* = 17)**	***t/*χ^2^*/U***	***p***	**ESE**
Age (years)	21.37 (1.64)	24.18 (5.68)	104.00	0.07	0.30
Sex			0.423	0.516	0.11
Male	8	9			
Female	11	8			
HLOE (years)	12.63 (1.38)	13.35 (1.94)	134.50	0.40	0.14
Shipley 2 IQ Score	107.53 (13.16)	104.3 (12.5)	0.75	0.46	0.25
BDI-II	4.32 (2.57)	4.12 (3.73)	0.19	0.85	0.10
PSQI	3.47 (1.31)	3.59 (1.12)	–0.28	0.78	−0.12
MAST	0.63 (0.68)	0.82 (1.24)	–0.59	0.56	−0.19
Dream Recall Frequency	4.68 (1.19)	0.24 (0.03)	0.000	<0.001^∗∗∗^	0.85
Recall Upon Awakening^a^			7.59	0.006^∗∗^	0.36
Yes	19	8			
No	11	21			

*For all variables except *Sex* and *Recall Upon Awakening* means are presented with standard deviations in parentheses. Chi-square statistic was computed for *Sex* and *Recall Upon Awakening*, and Mann–Whitney *U* for *Age, HLOE, and Dream Recall Frequency.* HFR, High Frequency Recall Individuals, LFR, Low Frequency Recall Individuals, ESE, effect size estimate (for *t*, Cohen’s *d;* for χ^2^, Cramer’s *V;* for *U, r*); HLOE, Highest Level of Education Completed in Years; BDI-II, Beck Depression Inventory-2nd Edition, PSQI, Pittsburgh Sleep Quality Index, MAST, Michigan Alcoholism Screening Test. *Dream Recall Frequency* relates to self-report of number of dream traces recalled per week; *Recall Upon Awakening* refers to whether a dream trace was recalled upon awakening in the sleep laboratory; ^*a*^there were 13 out of 72 missing dream reports (following the adaptation night); ^∗∗^*p* < 0.01; ^∗∗∗^*p* < 0.001.*

To demonstrate that individuals in the HFR and LFR groups did indeed have significantly different rates of DRF, we compared their recall frequency (a) gathered during the screening questionnaire and (b) upon awakening on the two study nights in the sleep laboratory. HFRs reported significantly more dreams than LFRs in both (a) and (b).

Because previous studies documented varying rates of DRF for the different sleep stages (Nielsen, 2000), we also investigated whether HFRs and LFRs differed in terms of the sleep stage prior to awakening. Chi squared analyses showed that there were no differences in awakenings from NREM 1, *X*^2^ = 0.75, *p* = 0.46, *V* = 0.11; NREM 2, *X*^2^ = 3.52, *p* = 0.07, *V* = 0.25; NREM 3, *X*^2^ = 1.93, *p* = 0.49, *V* = 0.18; REM sleep, *X*^2^ = 0.21, *p* = 0.76, *V* = 0.06; and waking (where participants were already awake when the experimenter entered to wake them up); *X*^2^ = 1.71, *p* = 0.25, *V* = 0.17.

### Between-Group Differences in WASO Percentage and the Number of Awakenings

Results confirm that individuals in the HFR group had a significantly higher percentage of WASO compared to the LFR group (see Table 2).

**TABLE 2:** Sleep architecture and sleep characteristics of the current sample (*N* = 36).

	**Group**				
	**HFR**	**LFR**			
**Variable**	**(*n* = 19)**	**(*n* = 17)**	***t/U***	***p***	**ESE**
Sleep Onset Latency	32.84 (20.65)	22.10 (14.85)	1.78	0.084	0.58
Sleep Efficiency	85.21 (11.45)	90.07 (5.45)	–1.60	0.120	0.52
WASO%	14.45 (10.95)	7.04 (3.74)	75.00	0.003^∗∗^	0.46^a^
NREM 1%	11.06 (4.87)	12.87 (7.51)	–0.87	0.393	0.28
NREM 2%	57.50 (15.42)	58.76 (7.73)	–0.306	0.762	0.10
NREM 3%	15.05 (7.57)	13.50 (4.95)	0.737	0.466	0.23
REM sleep%	13.31 (6.81)	14.99 (5.06)	–0.831	0.412	0.27
REM Onset Latency	143.50 (69.94)	174.64 (33.68)	–0.167	0.104	0.54
Total Awakenings	25.26 (10.79)	18.59 (6.41)	2.222	0.017^∗^	0.72
Stage 2 Awakenings	17.47 (9.95)	10.29 (4.82)	2.704	0.011^∗^	0.88
Stage 3 Awakenings	2.47 (0.91)	2.65 (1.77)	159.00	0.933	0.01
REM Awakenings	5.21 (3.43)	5.77 (4.12)	–0.441	0.662	0.15
Total REM Density	44.80 (27.40)	36.58 (15.47)	132.00	0.175	0.18

*For all variables means are presented with standard deviations in parentheses. HFR, High Frequency Recall individuals, LFR, Low Frequency Recall individuals; WASO%, Wake After Sleep Onset percentage; *t* Statistic computed for all variables, except for WASO%, Stage 3 Awakenings, and Total REM Density where Mann–Whitney *U* was computed as part of non-parametric analyses. ESE, Effect size estimate; ^*a*^Cohen’s *d* computed for all variables, with the exception of WASO%, Stage 3 Awakenings, and Total REM Density where *r* was computed; two-tailed analyses implemented for all variables, except for WASO%, Total Awakenings, and Total REM Density where *a priori* predictions were made. ^∗^*p* < 0.05; ^∗∗^*p* < 0.001.*

Our findings also revealed that, firstly, HFRs experienced significantly more total awakenings across the night when compared to the LFRs, and secondly, that there was no significant difference in the number of awakenings from REM sleep between groups (see Table 2).

However, surprisingly, individuals in the HFR group had significantly more frequent awakenings from NREM 2 sleep than individuals in the LFR group. The results show, in fact, that the difference in total awakenings between the two groups can be accounted for by the difference in awakenings from NREM 2 sleep and not from other sleep stages, where the number of awakenings were comparable.

In order to further elucidate this finding, we also investigated whether HFRs experience significantly more awakenings lasting ≥2 min from both REM and NREM 2 sleep compared to LFRs. Results showed that HFRs experienced significantly more awakenings lasting ≥2 min compared to LFRs, while no significant difference was found in relation to the number of ≥2 min awakenings from REM sleep (see Table 3). Finally, we also examined the dream recall rates from both REM sleep and NREM 2 sleep. We found a significant between-group difference in recall rates from NREM 2 sleep, while the recall rate from REM sleep was not significantly different (see Table 3).

**TABLE 3:** Rapid eye movement and NREM 2 Sleep Awakening and Recall Profile (*N* = 36).

	**Group**				
	**HFR**	**LFR**			
**Variable**	**(*n* = 19)**	**(*n* = 17)**	***t/U/*χ^2^**	***p***	**ESE**
Long REM Awakenings	0.26 (0.45)	0.71 (1.05)	130.00	0.23	0.27
Long NREM 2 Awakenings	2.68 (1.73)	1.24 (1.15)	2.92	0.006^∗∗^	0.98
REM Recall Rate^a^	50%	28.6%	0.63	0.59	0.22
NREM 2 Recall Rate^b^	81.3%	30%	6.83	0.015^∗^	0.51

*HFR, High Frequency Recall Individuals; LFR, Low Frequency Recall Individuals. For Long (≥2 minute) awakenings and Long N2 Awakenings means are presented with standard deviations, while Mann–Whitney *U* was computed for the former and *t* for the latter. Chi-square statistic was computed for *REM Recall Rate* and *NREM 2 Recall Rate.*^*a*^*REM Recall Rate* was based on 6 and 7 dream reports for HFRs and LFRs, respectively. ^*b*^*NREM 2 Recall Rate* was based on 16 dream reports from the HFR group and 10 from the LFR group. ESE, effect size estimate (for *t*, Cohen’s *d*; for *U, r*; for χ^2^, Cramer’s *V*). ^∗^*p* < 0.05. *^∗∗^p* < 0.01.*

### Between-Group Differences in REM Density

HFR individuals did not show significantly higher Total REM density compared to LFR individuals.

## Discussion

The present study had two aims. Firstly, we aimed to show that HFRs have a higher awakening profile (characterized by both time spent awake after sleep onset and the number of awakenings) compared to LFRs.

We found that, consistent with findings related to polysomnographic data from Eichenlaub et al. (2014a), HFRs spent significantly more time awake after sleep onset. Our findings also showed that HFRs had more frequent awakenings in comparison with LFRs. However, contrary to our prediction, HFRs experienced the majority of their awakenings from NREM 2 rather than REM sleep, while LFRs experienced similar amounts of awakenings during NREM 2 and REM sleep.

Secondly, via examining REM density as a proxy for dream production, we tested the prediction that HFRs would have higher REM density than LFRs. We did not find any between-group differences in this marker of dream production.

Regarding our first aim, the pattern of increased wakefulness after sleep onset and increased total number of awakenings experienced by HFRs fits well with the encoding and retrieval mechanisms postulated by the arousal-retrieval model. For example, our data supports the hypothesis that an increased number of awakenings leads to an increased number of opportunities for dream traces to be encoded. Furthermore, the longer periods of wakefulness following awakenings that HFRs experienced may also enhance the encoding of dream content from short-to long-term memory, according to the model. These results build upon existing evidence to provide support for the validity of the arousal-retrieval model of dream recall.

With regard to a mechanism underlying the arousal-retrieval model, Eichenlaub et al. (2014a) propose that increased wakefulness after sleep onset, as well as awakenings from sleep in general in HFRs, show heightened brain reactivity. This assertion is based on results from their event-related potential (ERP) study which revealed that HFRs respond more strongly to novel auditory stimuli during both wakefulness and sleep. The neurophysiological mechanism underlying this heightened brain reactivity to stimuli in HFRs is thought to be a P3a-like component, or P3a-like wave detected on electroencephalogram (EEG). The P3a-like component (a sub-component of the P300 wave), is strongly associated with orientation of attention to external stimuli (Friedman et al., 2001). It is accepted that the larger the P3a-like wave is, the stronger is the attention orientation response (Dominguez-Borras et al., 2008; Lv et al., 2010). HFRs exhibited larger P3a-like waves in response to novel auditory stimuli across vigilance states compared to LFRs. Eichenlaub et al. (2014a) postulate that a stronger attention orientation response to external stimuli in HFRs is one of the neurophysiological mechanisms underlying awakenings and longer periods of wakefulness after sleep onset in these individuals.

Interestingly, detection of a larger P3a-like wave in HFRs in response to novel stimuli (either a participant’s first name or an unknown first name presented randomly and rarely among pure tones), was not homogenous across sleep stages (Eichenlaub et al., 2014a). For example, at earlier latencies (which represent the attention-orientation response), larger P3a-like waves in HFR individuals in response to novel stimuli were detected most strongly during NREM 2 sleep. Put differently, HFRs exhibited the strongest attention-orientation response to novel stimuli during NREM 2 sleep.

A stronger attention-orientation response during NREM 2 sleep could serve as one potential mechanism underlying the awakening profiles of HFRs. We found significant between-group differences in relation to the number of awakenings, as well as awakenings lasting ≥2 min from NREM2 sleep. The latter finding is of particular significance since, according to the arousal-retrieval model of dream recall, increased duration of awakenings should lead to enhanced dream recall (Koulack and Goodenough, 1976). Indeed, Vallat et al. (2017a) found that the minimum time period required for awareness of/memory for dream traces is approximately 2 min. Finally, the significant between-group difference in relation to dream recall rates from NREM 2 sleep in this study further underscores the critical role of NREM 2 awakenings in enhanced dream recall in HFRs.

Regarding our second aim, we found that HFRs did not exhibit higher REM density than LFRs, and therefore were not likely to experience more dreaming during REM sleep. There are two viable explanations for this finding. One is that differences do exist, but they were undetected in this study. More specifically, it could be argued that the methods and measures employed in our study were not sensitive enough to detect between-group differences. However, this is an unlikely explanation as there were strong and significant correlations between the REM density parameters and affective variables in a yet unpublished study from our laboratory (van Wyk, unpublished). It is unlikely that the measure would be sensitive enough to detect significant results in one investigation but not in another. This favors the second explanation for the null findings, which is that between-group differences with regard to REM density do not exist in the current sample.

In light of this, we propose that between-group differences were lacking because, perhaps, REM dreaming is not of prime relevance in the current sample. For example, there were no between-group differences with regard to REM density, REM sleep%, REM sleep latency, the number of awakenings from REM sleep, the number of awakenings lasting ≥2 min from REM sleep, nor dream recall rates from REM sleep awakenings. Furthermore, at the time of writing (2019), new findings with regard to REM sleep and REM density based on the Eichenlaub et al. (2014a) data, were published (Vallat et al., 2017a). Researchers found no significant difference with regard to REM density values, nor between the number and length of awakenings from REM sleep. They concluded that higher DRF in the HFR group “could not be explained by the REM sleep hypothesis of dreaming” (Vallat et al., 2017a). It is important to note that we independently obtained results comparable to Vallat et al. (2017a), despite utilizing a different method of measuring REM density.

Another point that should be emphasized is that the studies by Eichenlaub et al. (2014a, b) and Vallat et al. (2017a), and the current study, all recruited healthy participants devoid of psychiatric symptoms and/or diagnoses. This is important as several changes in REM sleep parameters have been noted in the presence of psychopathology (Cartwright et al., 1998; Ellis et al., 2014; Medina et al., 2014). Therefore, one possible reason for failing to detect significant differences in any of the REM sleep parameters could be because a sample free from psychopathology was recruited in the present study and similar research.

### Limitations and Directions for Future Research

Although the focus of this study was primarily on studying the awakening profiles of HFRs in relation to increased DRF, it must be mentioned that there are several other factors that exert a potential influence on reported DRF. For example, Schredl (2018) postulates that there are both personality (e.g., openness to experience, interest in dreams), and physiological factors influencing DRF. One important physiological factor relates to sleep inertia that might impair an individual’s ability to successfully recall dream traces due to its effects on the functionality of the memory systems (Schredl, 2018). Interestingly, Vallat et al. (2017b) found that sleep inertia appears to be less pronounced in HFRs compared to LFRs; therefore, reduced sleep inertia could be one important physiological factor contributing to increased DRF in HFRs.

There are also other factors related to the micro and macro structure of sleep that were not investigated in the current study that need to be considered. For example, Kirov et al. (2015) have shown that an increase in sequential sleep stage transitions between NREM and REM sleep enhance awareness upon awakening and promotes explicit knowledge generation. These sleep-dependent cognitive processes hold the potential of significantly affecting successful dream recall. Furthermore, it is also important to consider neurophysiological markers other than the P3a wave (attention-orientation response) that is inferentially linked to increased DRF in HFRs. For example, Marzano et al. (2011) found that successful dream recall is preceded by reduced alpha oscillation in the temporo-parietal areas. Future studies should assess whether there are morphological differences in alpha oscillations in the temporo-parietal areas in HFRs when compared to LFRs.

Another important avenue of investigation relates to increasing LFRs’ DRF experimentally and re-assessing their sleep architecture to see if there are any changes in their awakening profiles. This could also elucidate state/trait differences related to DRF.

Finally, future studies should aim to replicate results from the REM density analyses in the current study using the same methodology in a comparable experimental design. This would elucidate whether or not the REM density measurement methodology served as a limitation in our study, and it would speak to its validity as a robust way of measuring REM density in future.

## Conclusion

The current study provides empirical support for the arousal-retrieval model of dream recall by demonstrating that HFRs spent significantly more time awake after sleep onset compared to LFRs. In addition, we showed that HFRs experience a significantly higher, and longer, number of awakenings, from NREM 2 sleep. Interestingly, none of the REM sleep parameters reached significance, including REM density which serves as a possible marker of dream production. Taken together, our findings are consistent with Vallat et al. (2017a) conclusion that inter-individual variability in DRF in healthy HFRs and LFRs cannot be explained by the REM hypothesis of dreaming. Instead, the data indicates that NREM sleep, and more specifically, awakenings from NREM2 sleep, are of particular importance in relation to increased DRF in healthy HFRs.

## Data Availability Statement

The datasets generated for this study are available on request to the corresponding author.

## Ethics Statement

The studies involving human participants were reviewed and approved by the Research Ethics Committee, Department of Psychology, University of Cape Town and Department of Student Affairs, Faculty of Humanities, University of Cape Town. The participants provided their written informed consent to participate in this study.

## Author Contributions

MW, MS, and GL conceptualized the study and provided input for writing the manuscript. MW was responsible for the data collection and analyses.

## Conflict of Interest

The authors declare that the research was conducted in the absence of any commercial or financial relationships that could be construed as a potential conflict of interest.
